# Parent-of-Origin Effects in 15q11.2 BP1-BP2 Microdeletion (Burnside-Butler) Syndrome

**DOI:** 10.3390/ijms20061459

**Published:** 2019-03-22

**Authors:** Kyle W. Davis, Moises Serrano, Sara Loddo, Catherine Robinson, Viola Alesi, Bruno Dallapiccola, Antonio Novelli, Merlin G. Butler

**Affiliations:** 1 Lineagen, Inc., Salt Lake City, UT 84109, USA; mserrano@lineagen.com (M.S.); k.mullin.robinson@gmail.com (C.R.); 2 Laboratory of Medical Genetics, Bambino Gesù Children’s Hospital, IRCCS, Rome 00165, Italy; sara.loddo@opbg.net (S.L.); viola.alesi@opbg.net (V.A.); bruno.dallapiccola@opbg.net (B.D.); antonio.novelli@opbg.net (A.N.); 3 Departments of Psychiatry & Behavioral Sciences and Pediatrics, University of Kansas Medical Center, Kansas City, KS 66160, USA; mbutler4@kumc.edu

**Keywords:** 15q11.2 BP1-BP2 microdeletion (Burnside-Butler) syndrome, imprinting, parent-of-origin effects, phenotype-genotype correlation, autism, developmental delays, motor delays

## Abstract

To identify whether parent-of-origin effects (POE) of the 15q11.2 BP1-BP2 microdeletion are associated with differences in clinical features in individuals inheriting the deletion, we collected 71 individuals reported with phenotypic data and known inheritance from a clinical cohort, a research cohort, the DECIPHER database, and the primary literature. Chi-squared and Mann-Whitney U tests were used to test for differences in specific and grouped clinical symptoms based on parental inheritance and proband gender. Analyses controlled for sibling sets and individuals with additional variants of uncertain significance (VOUS). Among all probands, maternal deletions were associated with macrocephaly (*p* = 0.016) and autism spectrum disorder (ASD; *p* = 0.02), while paternal deletions were associated with congenital heart disease (CHD; *p* = 0.004). Excluding sibling sets, maternal deletions were associated with epilepsy as well as macrocephaly (*p* < 0.05), while paternal deletions were associated with CHD and abnormal muscular phenotypes (*p* < 0.05). Excluding sibling sets and probands with an additional VOUS, maternal deletions were associated with epilepsy (*p* = 0.019) and paternal deletions associated with muscular phenotypes (*p* = 0.008). Significant gender-based differences were also observed. Our results supported POEs of this deletion and included macrocephaly, epilepsy and ASD in maternal deletions with CHD and abnormal muscular phenotypes seen in paternal deletions.

## 1. Introduction

The 15q11.2 BP1-BP2 microdeletion syndrome (or Burnside-Butler syndrome; OMIM # 615656) is a neurodevelopmental disorder with clinical findings reported in hundreds of individuals [1,2]. This condition includes the deletion of four genes thought to be nonimprinted (*TUBGCP5, CYFIP1, NIPA1, NIPA2*), located between two distinct proximal 15q11.2 breakpoints (BP1 and BP2) and separated by about 500 kilobases (kb). Summarized findings from a large cohort of patients presenting for genetic services found that 0.41% of patients (69 of ~17,000) had a deletion of the proximal 15q11.2 BP1-BP2 region [3]. In a review of over 10,000 clinically affected individuals tested with ultra-high-resolution chromosome microarrays, the 15q11.2 BP1-BP2 microdeletion was the leading cytogenetic finding of those presenting with autism spectrum disorder (ASD) alone or ASD and other clinical features [4]. 

This condition can present with a wide range of clinical findings including cognitive deficits, language and/or motor delays, ASD, behavioral disturbances, poor coordination, ataxia, attention disorders, seizures, and dysmorphic or congenital anomalies [2]. Psychiatric findings can include schizophrenia, obsessive compulsive disorder, and oppositional defiant disorder. Dyscalculia, dyslexia and structural brain changes in both grey and white matter have been reported commonly in individuals with this deletion syndrome [5]. 

About 80% of children identified with the 15q11.2 BP1-BP2 microdeletion inherit it from a parent, who may or may not be clinically affected. Therefore, this susceptibility locus shows incomplete penetrance with variable expressivity. Approximately 30% of the parents ascertained through genetic testing with a clinically affected child due to the 15q11.2 BP1-BP2 microdeletion will have clinical findings or involvement [2]. 

Deletions can range from approximately 320 kb to 500 kb, though all four genes within this 320-kb region are highly conserved and, when disturbed, are associated with or cause neurological, motor, intellectual, and behavioral problems. For example, specific missense variants of the *NIPA1* (non-imprinted in PWS/AS 1; OMIM # 608145) gene are known to cause autosomal dominant hereditary spastic paraplegia and postural disturbance; repeat expansions have recently been associated with amyotrophic lateral sclerosis [6,7]. The *NIPA1* protein is known to mediate magnesium transport and is highly expressed in the brain [8,9]. The *NIPA2* (non-imprinted in PWS/AS 2; OMIM # 608146) gene is also involved in magnesium transport and childhood absence epilepsy reported in a Han Chinese cohort [10]. However, this association has not been replicated in other cohorts and pathogenicity is unclear [11]. The third gene in the 15q11.2 BP1-BP2 region is *TUBGCP5* (tubulin gamma complex associated protein 5; OMIM # 608147) and is associated with attention-deficit hyperactivity disorder (ADHD) and obsessive-compulsive behavior. A recent publication associated biallelic loss of this gene with primary microcephaly [12]. Lastly, the *CYFIP1* (cytoplasmic fragile X mental retardation 1 *FMR1* interacting protein 1; OMIM # 606322) gene encodes a protein that interacts with FMRP, the protein produced by the *FMR1* (Fragile X Mental Retardation 1; OMIM # 309550) gene and in which triplet-repeat expansion causes fragile X syndrome, the most common cause of inherited cognitive disabilities in families [13]. The *CYFIP1* gene also interacts with the protein from the *RAC1* (RAS-related c3 botulinum toxin substrate 1; OMIM # 602048) gene, disruption of which causes an autosomal dominant form of intellectual disability [14]. Recent research has also shown that reduced *CYFIP1* expression leads to dysregulation of schizophrenia- and epilepsy-associated gene networks [15]. Mouse models have found that *CYFIP1* regulates development, function, and plasticity of presynaptic neurons [16].

Although these genes have previously been reported as non-imprinted, recent research found a methylated site within this chromosome 15 region in human DNA samples [17] and unequal gene expression in mice [18]. Using blood samples from individuals with maternal or paternal disomy 15, a maternally methylated CpG island near the promoter of the *TUBGCP5* gene was identified [17]. Additionally, mouse models heterozygous for maternal *or* paternal loss of the *CYFIP1* gene found unequal parental expression in the cortex, with different behavioral outcomes depending on parental inheritance patterns [18]. 

Several possible explanations may exist for the incomplete penetrance and variable expressivity observed in this condition. First, clinically affected individuals may have two hits, such as the cytogenetic microdeletion and a pathogenic variant of one or more of the genes in the 15q11.2 BP1-BP2 region or other developmentally important genes, while the clinically unaffected parent may have only the microdeletion. Second, a parent or their child may be mildly affected and not seek medical attention (i.e. ascertainment bias). A third possibility is that unequal parental expression of one or more genes causes specific phenotypes.

Given new data about *CYFIP1* gene expression and the maternally methylated region found near the *TUBGCP5* gene promoter, supported by previous expression studies showing parental bias in this region from lymphoblasts [19], deletions of these genes may show a parent-of-origin effect (POE). For further investigation, we sought to determine if such an effect could be observed by analyzing reported clinical features in probands and the specific parental inheritance patterns of the deletion. 

## 2. Results

### 2.1. Cohort Characteristics

Our cohort included 71 individuals, mostly male probands (*N* = 42, 1.4 male-to-female ratio), had an average age of testing at 6.9 years for males and 9.4 years for females (7.9 years among all individuals), and most were unrelated (i.e., not siblings; *N* = 55, 77%). This age distribution and male-to-female ratio is similar to a previous study of 52 individuals with a 15q11.2 BP1-BP2 microdeletion [20], which found a 1.7 male-to-female ratio and average age of testing at 8.6 years. Statistically significant differences were noted between male and female carriers of the 15q11.2 BP1-BP2 microdeletion using two-tailed *t*-tests, as males had significantly more clinical features as well as non-physical features (Table 1). 

However, within paternally- and maternally-inherited deletions, the male-to-female ratio differed from that of the full group. For paternally-inherited deletions, we observed a 1.1 male-to-female ratio (19 males vs. 18 females), whereas in maternally-inherited deletions we observed a 2.1 male-to-female ratio (23 males vs. 11 females). Using these ratios in chi-squared testing, we found that there is a statistically significant difference in the male-to-female ratio between paternally- and maternally-inherited deletions (*p* = 0.03). 

Using chi-squared testing with the 1.7 male-to-female ratio reported by Vanlerberghe et al. [20] to derive an “expected” ratio of males-to-females within each parental deletion group, we found that neither male-to-female distribution was significantly different from 1.7. For paternal deletions, we used our observed ratio of 1.1 (19 males and 18 females) versus an expected ratio of 1.64 (23 males and 14 females, *p* = 0.48). For maternal deletions, we again used our observed ratio of 2.1 (23 males vs. 11 females) versus and expected ratio of 1.62 (21 males vs. 13 females, *p* = 0.8).

Loss of the 15q11.2 BP1-BP2 region was slightly more often paternally than maternally inherited (*N* = 37 and 34, respectively). No differences were observed regarding the parental origin of the deletion in relation to the proband’s average age at genetic testing, the total number of clinical features, total physical symptoms, or total non-physical symptoms (Table 2). When analyzing our cohort by specific clinical features, the most common findings were speech and motor delays (*N* = 35, 49% for both) followed by facial dysmorphisms (*N* = 30, 42%). See Table 3 for the frequency of individual clinical features found in this set of probands.

### 2.2. Differences in Clinical Features by Parent-of-Origin of the 15q11.2 BP1-BP2 Microdeletion

When analyzing for differences using the entire cohort (*N* = 71), we found statistically significant differences in several clinical features based on the parental inheritance of the 15q11.2 BP1-BP2 microdeletion (Table 4). Using chi-squared analyses, we found paternal but not maternal deletions to be significantly associated with congenital heart disease (CHD; 22% vs. 0%, *p* = 0.004). However, maternally inherited deletions were significantly associated with macrocephaly (15% vs. 0%, *p* = 0.016) and ASD (29% vs. 8%, *p* = 0.02). 

Several clinical features remained significantly associated with specific parental inheritance when controlling for sibling sets and other genetic variants (a VOUS). In the cohort without sibling sets (*N* = 55), CHD was still significantly more likely in individuals with paternal deletions compared with maternal deletions (19% vs. 0%, *p* = 0.013); muscle-related clinical findings (e.g., hypotonia) were also associated with paternal deletions (50% vs. 24%, *p* = 0.047). Maternal deletions were significantly associated with macrocephaly (17% vs. 0%, *p* = 0.026) and associated with epilepsy (34% vs. 12%, *p* = 0.046). In the cohort without sibling sets and/or individuals with an additional VOUS (*N* = 44), the association between CHD and paternal deletions became non-significant (10% vs. 0%, *p* = 0.113), while muscle-related clinical features in paternal deletions strengthened (55% vs. 17%, *p* = 0.008); epilepsy remained significantly associated with maternal deletions (42% vs. 10%, *p* = 0.019).

Mann-Whitney U-test revealed marginally statistically significant differences among the entire cohort between maternally and paternally inherited deletions in the non-physical features group variable. Maternally inherited deletions had a higher median number of clinical features than paternally inherited alleles (*p* = 0.04). However, this difference was not observed when removing sibling sets or probands with an additional VOUS.

### 2.3. Differences in Clinical Features between Proband Gender 

Given our unique and granular dataset, we also did exploratory analyses to determine if there were differences in clinical features based on an individual’s gender, as a previous report found substantial differences in neurodevelopmental features between males and females with various genetic conditions [21]. Using chi-squared analyses, several statistically significant differences emerged in clinical features when analyzing the entire cohort between males and females with 15q11.2 BP1-BP2 microdeletions (Table 4). 

When using the entire cohort (*N* = 71), we found males were significantly more likely than females to have motor delays (64% vs. 28%, *p* = 0.002), a behavioral phenotype (64% vs. 31%, *p* = 0.006), and any type of developmental delay (79% vs. 48%, *p* = 0.008). Additionally, learning difficulties and psychiatric diagnoses were marginally associated with males (both clinical features: 35% vs. 17%, *p* = 0.058). Only epilepsy was significantly associated with females (38% vs. 14%, *p* = 0.022).

In the cohort without sibling sets (*N* = 55), males were still significantly more likely than females to have motor delays (64% vs. 32%, *p* = 0.021), while males were marginally more likely to have any type of developmental delay (79% vs. 55%, *p* = 0.057) and receive genetic testing at a younger age (5.4 vs. 9.9 years of age, *p* = 0.018). In the cohort without sibling sets and/or individuals with an additional VOUS (*N* = 44), males were still significantly more likely than females to have motor delays (68% vs. 32%, *p* = 0.017), any type of developmental delay (76% vs. 47%, *p* = 0.05) and receive testing at a younger age (3.5 vs. 4.9 years of age, *p* = 0.05).

## 3. Discussion

Our study identified differences in specific clinical features depending on the parental inheritance of a 15q11.2 BP1-BP2 microdeletion. Several differences remained significant when removing sibling sets and other genetic variants from the analyses and provide evidence that POE exists in this deletion syndrome. Additionally, we observed an unequal male-to-female ratio between maternal verse paternal deletions. Neither paternal nor maternal deletions appeared to cause a more “severe phenotype” (i.e., more clinical features); however, unequal distribution of clinical features were found providing evidence for POE. 

The basis for POE in this condition is buttressed by several pieces of data. First, a recent publication analyzing potential methylated regions in individuals with various regions of uniparental disomy found a maternally methylated region near the promoter of the *TUBGCP5* gene [17]. This methylated segment may act on the *TUBGCP5* gene, other genes in this cytogenetic region, or may act in a tissue-specific manner. However, previous studies of expression using blood samples have not found altered expression for genes within this deletion [22,23,24]. But, a second line of evidence supportive POE uses mouse models heterozygous for loss of the *CYFIP1* gene for either the maternal or paternal allele, which showed unequal and significant differences in expression in the brain cortex, which was correlated with differences in observed behaviors [18]. Another intriguing line of evidence is the differences in male-to-female ratios in maternal versus paternal deletions (2.1 vs. 1.1). Lastly, several genes in this broader region of chromosome 15 are methylated and known to cause genetic conditions with POEs, including Schaff-Yang syndrome (OMIM # 615547) with paternal loss of the imprinted *MAGEL2* gene; Angelman syndrome (OMIM # 105830) with maternal loss of the imprinted *UBE3A* gene; Prader-Willi syndrome (OMIM # 176270) due to paternal loss of imprinted genes and transcripts in the 15q11-q13 region such as *SNRPN;* and central precocious puberty 2 (OMIM # 615346) with paternal loss of the imprinted *MKRN3* gene. Given previous data, analysis of the literature and our findings, POE seems likely to exist for the 15q11.2 BP1-BP2 microdeletion with involvement of one or more genes within this region as described below. 

Which gene or genes within the 15q11.2 BP1-BP2 region are undergoing POE is unknown at this time, as all four genes within this ~500 kb region are highly conserved, apparently biallelically expressed (at least in blood) and not thought to be imprinted [24,25]. However, Bittel and colleagues [19] reported unequal parental expression bias compared with controls for the SHGC-32610 transcript located proximal to the D15S1035, a standard marker in the 15q11.2 BP1-BP2 region at that time and significantly increased in expression in lymphoblastoid cell lines established from individuals with Prader-Willi syndrome having either maternal disomy 15 or the paternal 15q11-q13 deletion. Loss of the 15q11.2 BP1-BP2 region causes more severe behavioral symptoms and learning difficulties in individuals with Prader-willi syndrome or Angelman syndrome [1,24,26].

Of the genes in this region, certain pathogenic variants in the *NIPA1* gene causes an autosomal dominant form of spastic paraplegia and triplet repeat expansion within this gene is associated with a higher risk for amyotrophic lateral sclerosis [6,7]. Haploinsufficiency of this gene has not been reported to cause these conditions. Variants in the *NIPA2* gene are reported to cause childhood absence epilepsy [10,27]. Both the *NIPA1* and *NIPA2* genes regulate magnesium transport in neurons [9,27]. The *TUBGCP5* gene is expressed highly in the subthalamic nuclei of the brain and plays a role in formation and function of the centrosome [12,28]. A recent study proposed that biallelic loss of this gene may cause a form of microcephaly, as a rare missense variant in *TUBGCP5* was identified in *trans* with a microdeletion of 15q11.2 BP1-BP2 [12]. Variants in this gene have also been associated with ADHD and obsessive-compulsive disorder [29]. Lastly, the *CYFIP1* gene encodes a protein with multiple actions in the cell, including participating in maturation and stabilization of dendritic spines and organization of the actin cytoskeleton [30]. The CYFIP1 protein interacts with the FMR1 protein (and other proteins) to control neuronal mRNA transcription and translation [15]. Reduced expression of *FMR1* causes fragile X syndrome, the most common cause of familial intellectual disability [13]. Haploinsufficiency of *CYFIP1* can cause similar symptoms to fragile X syndrome in mice [25,31]. Additionally, the CYFIP1 protein is also a member of the WAVE regulatory complex, which plays a role in actin polymerization [32]. Reduced expression of the *CYFIP1* gene in model organisms and human blood samples correlates with reduced mRNA for WAVE regulatory complex members. Additionally, the *CYFIP1* gene has been found to be differentially expressed in the brain depending on the stage of embryonic development in mice, with the highest expression in the cortex and cerebellum [30].

Current and emerging evidence points to altered expression of the *CYFIP1* gene as the leading candidate for neuronal phenotypes and thus becomes a candidate for a potential POE. In the previous mouse model study of *CYFIP1* gene haploinsufficiency, maternal loss of *CYFIP1* (leading to only paternal allele expression) showed significantly higher expression in the mouse cerebral cortex than paternal loss of *CYFIP1.* Maternal loss and paternal expression of *CYFIP1* was 55%-57% relative to wild type, while paternal loss and maternal expression was 48%–52% (*p* = 0.03) [18]. No other differences were found in expression for other brain tissues studied, including the hippocampus, amygdala, and cerebellum. More recently, *CYFIP1* gene expression in murine brain tissue and expression patterns are dependent on the POE of the deletion. For example, paternal loss of the *CYFIP1* gene was associated with lower protein expression in the hypothalamus, while maternal loss was associated with lower expression in the nucleus accumbens [33]. In this study, the effect of certain *CYFIP2* variants was tested and the expression patterns were dependent on the gender of the mice. Given these studies, there is evidence that mild preferential expression for the paternal *CYFIP1* allele exists (at least in certain tissues), which would be expected if a maternally methylated region were acting in *cis* on the *CYFIP1* gene.

Previous work using human neural progenitor cells has found that reduced *CYFIP1* expression caused dysregulation in schizophrenia- and epilepsy-associated gene networks [15]. However, neither expression patterns nor a POE were studied. The *CYFIP1* gene is known to interact with the WAVE regulatory complex and proteins from two genes: *RAC1,* disruption of which causes an autosomal dominant form of intellectual disability, and *FMR1*, the causative gene for fragile X syndrome [14,16,31,34]. The CYFIP1-FMRP protein complex has been found to control both transcription and translation of mRNAs in neuronal cells [15]. Additionally, previous studies have noted that *CYFIP1* haploinsufficiency can generate features of the fragile X syndrome phenotype in mice [25,31], while a subgroup of individuals with fragile X syndrome have a Prader-Willi-like phenotype, but no specific cause for these phenotypes is known [35].

In terms of specific clinical features showing a potential POE, we found that CHD, macrocephaly, ASD, epilepsy and muscle-related phenotypes were statistically associated with a deletion from a specific parent. Although selection bias is a significant concern, and this region is known as a susceptibility region, previous work suggested that a POE does exist in CHD involving the 15q11.2 BP1-BP2 microdeletion as well as in well-characterized imprinting disorders such as Prader-Willi syndrome resulting from a paternal 15q11-q13 deletion or maternal uniparental disomy 15 [36]. Kuroda and colleagues [36] found seven individuals with CHD had paternal inheritance of the 15q11.2 BP1-BP2 microdeletion while only one individual with CDH was found when the deletion was from the mother. (Our dataset included two of these seven individuals with paternal deletions.) Our data further buttress this finding, as CHD was reported exclusively in individuals with paternally inherited deletions (8 vs. 0 including siblings, 5 vs. 0 omitting siblings). Although our sample size was too small to detect a statistical difference when omitting sibling sets and individuals with a VOUS, there were two individuals with CHD and paternal deletions and zero with maternal deletions. 

Interestingly, the *CYFIP1* gene has the highest expression of the four genes in this chromosomal region in both heart muscle and vasculature [37]. For example, expression in four types of cardiac tissue from the Gene-Tissue Expression Project (GTEx) showed that, relative to other genes in this deletion, *CYFIP1* was expressed 2.9-8.8x higher in the aorta, 2.2-8.2x higher in the coronary artery, 1.1-5.0x higher in the left ventricle, and 1.2-4.2x higher in the atrial appendage. A previous study found that CHD was highly enriched in those individuals with the 15q11.2 BP1-BP2 microdeletion but the phenotype was not consistent involving both heart muscle and vasculature [38]. This lack of a phenotypic pattern appears consistent with the *CYFIP1* expression data, such that dysregulated expression could result in multiple different types of CHD. 

A similar pattern for *CYFIP1* gene expression in heart muscle was also observed in the GTEx data for skeletal muscle, where *CYFIP1* has the second highest expression, at 1.8x higher than *TUBGCP5* and 6.2x higher than *NIPA1*, while being slightly lower than *NIPA2* expression. CHD and muscular phenotypes were both associated with paternally inherited 15q11.2 BP1-BP2 microdeletions, while mouse models showed a preference for paternal *CYFIP1* expression in various parts of the brain. Possibly, the paternal *CYFIP1* allele is preferentially expressed in other tissues as well, such as the heart or skeletal muscle and vasculature. 

Our association for maternal deletions with ASD, macrocephaly, and epilepsy are intriguing. Only epilepsy remained significantly associated with maternal deletions when omitting siblings and probands with a VOUS finding. However, it should be noted that we lost statistical power from our small sample size to determine if a difference was present in these features when omitting these probands. Although our results were not statistically significant, ASD and macrocephaly were enriched in individuals with a maternal deletion (21% vs. 5% and 13% vs. 0%, respectively). This association may hold when omitting siblings and probands with a VOUS, but a larger sample size is needed. 

Interestingly, clinical features of fragile X syndrome in humans can include the three features associated with maternal 15q11.2 BP1-BP2 microdeletions (ASD, seizures, and macrocephaly) [39]. *RAC1*-related intellectual disability also includes macrocephaly and one individual was reported to have ASD. One possibility for the associated phenotypes with a maternal POE is the maternal *CYFIP1* gene allele is preferentially expressed in different brain tissue(s) and a loss of the maternal allele is more detrimental than loss of the paternal allele in these tissues, which may somehow disrupt *FMR1* and/or *RAC1* activities or the WAVE regulatory complex. In one study using a mouse model, there was evidence for maternal expression of *CYFIP1* in the nucleus accumbens [33]. Further, Abekhoukh and colleagues [32] noted that previous studies found inconsistent neural spine phenotypes when observing *CYFIP1*-deficient mice. A POE was not assessed in either of these studies and could potentially explain these differences. Lastly, we cannot rule out that this association between maternal deletions and ASD, epilepsy, and macrocephaly could be spurious, influenced by ascertainment bias, or both.

Differences in clinical features between males and females has been noted in other neurodevelopmental conditions [21]. Our analyses between males and females are notable because we applied statistical testing to the distribution of clinical features based on gender, which has not been done in a previous, large case series [20]. Our findings indicate that females were more likely to have epilepsy, while males were more likely to have either motor or developmental delays. These differences may be due to distinct biological differences; however, it is also possible that these represent ascertainment bias and females received a medical examination and genetic testing when a more “severe” symptom was present, such as epilepsy, while milder clinical features may have been ignored or thought less important to investigate. Similarly, males with a developmental delay may have been more likely to receive an examination.

Several other points have been discussed in the literature regarding 15q11.2 BP1-BP2 microdeletions. First, one study suggested that this condition may show a “two-hit” model, such that individuals with additional genomic variants that impact neurodevelopment, as well as a 15q11.2 BP1-BP2 microdeletion, may be more likely to have clinical features or perhaps have more clinical features than individuals with only a 15q11.2 BP1-BP2 microdeletion [40]. Although we had a large sample size, the number of individuals with additional genomic alterations (*N* = 12) was too limited to determine if phenotypic differences existed between individuals with another genomic alteration; also, no individuals were reported to have a second alteration within one of the four genes in the 15q11.2 BP1-BP2 region. However, sequence variants were not routinely assessed in our cohort. Second, a recent paper identified a statistically significantly enrichment of the 15q11.2 BP1-BP2 microdeletion in three individuals with gender dysphoria (3 of 69 birth-assigned females; 4.3%) [41], suggesting that this deletion may influence gender identity. Of the 71 probands in our study, none were specifically noted to have gender dysphoria or other disorders of gender development. While we cannot rule out this deletion is associated with gender dysphoria, our data do not support the possibility that this is an additional clinical feature. 

Lastly, we can assess the rate at which parents were identified with clinical features of 15q11.2 BP1-BP2 microdeletion syndrome. Previous studies have estimated that approximately 30% of parents are affected [2]. We found that when phenotype information was available, and in unrelated individuals to avoid double-counting parents with multiple children in our cohort, approximately 38% of parents (19 of 50) were found to have one or more clinical feature. Because some studies did not report on parental clinical features and other papers may not have assessed parental clinical or developmental history, we restricted our analysis to the two cohorts of individuals that represent a “high-confidence” group for detailed phenotyping. These cohorts included probands and their parents assessed by two different geneticists from the ongoing study of chromosomal 15 abnormalities and the group identified during routine clinical work-up (*N* = 22 when omitting sibling sets). In these groups, 50% of parents (11 of 22) were affected with one or more clinical feature. In contrast, the frequency reported in individuals from the primary literature was approximately 26% (7 of 27). Although our 50% rate may represent ascertainment bias of the parent’s child, it is likely that more parents are more often affected (albeit mildly) and previous studies were not sensitive to this possibility or to the full phenotypic spectrum.

Our study had several strengths, including the large sample size, use of statistical analyses, granular analyses of clinical features, investigation of gender-based differences and parental penetrance, and our ability to control for other potentially confounding variables in clinical variability, such as siblings and additional genomic alterations. The authors encourage additional studies, both clinically and by genomic characterization to delineate this emerging microdeletion syndrome to gain a better understanding of the collection of clinical findings and their causation, specifically in view of our evidence presented on parent-of-origin effects.

Limitations in this study include likely ascertainment bias, variable quality in phenotypic information from disparate sources, that uncharacterized genes within larger BP1-BP2 deletions may play a role in one or more phenotype(s), and the fact that individuals in this cohort were not evaluated by the same observer(s). Indeed, the average number of reported symptoms in the probands assessed by the two geneticists were 5.6, while the average number of symptoms in probands from the primary literature was 3.4. Additionally, it is possible that some of these associations are spurious and will not be consistent in follow-up studies. Further studies analyzing POEs in this condition are warranted, especially gene expression studies and the presence of a maternally methylated region near the *TUBGCP5* gene. Lastly, it is possible, though unlikely, that a small percentage of individuals in this study were reported in multiple sources, such as the online database DECIPHER and later reported in a paper in the primary literature. Given the nature of this research, we cannot be absolutely sure we did not double-count individuals in our analyses. Regardless, this is most likely a small risk and unlikely to impact the main findings. Additional research with a deeply phenotyped cohort assessed by the same observer(s) would be helpful. Finally, many of these individuals were not reported to have undergone a next generation sequencing study (e.g., exome), and therefore, a second variant associated with neurodevelopmental findings cannot be ruled out and will require further studies. 

## 4. Materials and Methods 

We collected 71 reported individuals with known parental inheritance of a 15q11.2 BP1-BP2 microdeletion from four sources: (1) the medical literature (*N* = 43) [22,23,36,42,43,44,45,46,47,48,49,50], (2) the DECIPHER database (*N* = 1) [51], 3) a cohort of patients with 15q11.2 BP1-BP2 microdeletion syndrome obtained during routine genetic diagnostic procedures (*N* = 11), and 4) a genetics study of chromosome 15 abnormalities, including 15q11.2 BP1-BP2 microdeletions (*N* = 16). This study of chromosome 15 abnormalities was approved by the University of Kansas Medical Center IRB to study genotype-phenotype correlations (FWA#: 00003411). As all individuals in this study were either previously published or de-identified data was provided from families who gave consent to share data, our study did not require IRB approval or a waiver. 

In order to standardize this cohort for analysis of the potential POEs, we omitted individuals with an additional known, abnormal genetic diagnoses (e.g., Williams syndrome) and reports of *de novo* 15q11.2 BP1-BP2 microdeletions. All clinical features were categorized as a specific feature (e.g., microcephaly) when possible or a general clinical finding if the symptom noted was non-specific (e.g., “delays” versus speech delay or motor delay). We also grouped clinical features into overarching categories. Individual and grouped symptom-related variables were coded categorically (present vs. absent). For example, the variable “Psychiatric diagnosis” was classified as being present if an individual had specific diagnoses, such as anxiety or obsessive-compulsive disorder. Similarly, the variable “Behavioral differences” included individuals with “difficult” or “odd” behaviors, such as aggression or skin picking. 

The four grouped variables included: (1) “any behavioral features” and included the categories of ASD diagnosis, psychiatric diagnoses, and any behavioral difference; (2) “any physical features”, which included CHD or malformations, short stature, micro/macrocephaly, and dysmorphisms; (3) “non-physical features” and included developmental delays, muscular features, intellectual disability, epilepsy, ASD, learning difficulties, psychiatric diagnoses, and behavioral differences and lastly (4) “any delays” which included speech delays, motor delays, global developmental delays, and any mention of non-specific delays. The only variable that was not categorical was “Total clinical features”, which added the described clinical features for an individual into a continuous variable. For example, if an individual was noted to have dysmorphic facial features, obsessive-compulsive disorder, ADHD, and ASD, this would count as three total clinical features because these features fall into three general categories (dysmorphisms, psychiatric diagnoses, and an ASD diagnosis).

In primary analyses, we used chi-squared tests to ascertain differences between individuals reported with a specific clinical symptom and the parent of origin for the deletion (maternal vs. paternal). Mann-Whitney U-tests were used to determine differences in grouped clinical features, as the distribution of these variables was non-normal. In sub-analyses, we performed chi-squared and Mann-Whitney U-tests on groups that omitted (1) sibling sets and (2) sibling sets and individuals with one or more additional VOUS. This was done to control for the fact that (1) shared genetic variants between siblings that may cause or contribute to certain clinical features and (2) a VOUS finding may be pathogenic and also cause or contribute to clinical features. In secondary analyses, we also used two-sided *t*-tests to determine if cohort characteristics differed between gender (e.g., age of diagnosis), as well as chi-squared to tests differences in specific clinical findings and Mann-Whitney U-test to determine differences between grouped symptoms (e.g., physical features). All findings were considered significant when *p* ≤ 0.05.

Lastly, these analyses were conceived and conducted solely by the authors; the original contributors to the DECIPHER Database bear no responsibility for this analysis or interpretation. 

## 5. Conclusions

The findings from the literature and survey reports add further clinical evidence to the previous molecular findings that the 15q11.2 BP1-BP2 microdeletion (Burnside-Butler) syndrome may exhibit POEs. Several gender-based differences in clinical features were reported in individuals with the 15q11.2 BP1-BP2 microdeletion. These findings, if replicated, may help prognosis and in counseling families identified with a 15q11.2 BP1-BP2 microdeletion to further expand the clinical phenotype of this emerging syndrome, now recognized as the most common cytogenetic finding in those presenting with ASD with or without congenital anomalies and developmental delays.

## Figures and Tables

**Table 1:** Descriptive statistics of clinical features by gender in those with the 15q11.2 BP1-BP2 microdeletion.

Variable	Female Probands (*N* = 29)				Male Probands (*N* = 42)				
	Avg	SD	Med	Range	Avg	SD	Med	Range	*p*
Age (Years)	9.4	8.2	7.0	0.08–27	6.9	5.2	6.0	0.25–24	0.130
Total Symptoms	3.4	2.4	2.0	1–10	4.9	2.2	4.5	1–10	0.020
Physical Features	0.9	1.0	1.0	0–3	1.0	0.9	1.0	0–3	0.610
Non-physical Features	2.5	2.4	2.0	0–9	3.95	2.1	4.0	0–8	0.009

Avg: average; Med: median; *t*-test; *p-*values (significance *p* < 0.05); compares differences in average age or symptoms in male and female probands with the 15q11.2 BP1-BP2 microdeletion.

**Table 2:** Descriptive statistics of parental inheritance of individuals with the 15q11.2 BP1-BP2 microdeletion.

Variable	Maternal (*N* = 34)				Paternal (*N* = 37)				
	Avg	SD	Med	Range	Avg	SD	Med	Range	*P*
Age (Years)	8.7	7.1	6.5	1.5–27	7.1	6.2	5.3	0.08–24	0.33
Total Symptoms	4.7	2.2	5.0	1–9	3.9	2.8	3.0	1–10	0.18
Physical Features	0.8	0.9	1.0	0–3	1.0	0.9	1.0	0–3	0.35
Non-physical Features	3.9	2.1	4.0	0–8	2.9	2.5	2.0	0–9	0.07

Avg: average; Med: median; *t*-test; *p-*values (significance *p* < 0.05); compares differences in average age or symptoms based on parental inheritance of the 15q11.2 BP1-BP2 microdeletion.

**Table 3:** Frequency of clinical features in the 71 probands with the 15q11.2 BP1-BP2 microdeletion.

Clinical Feature	Percentage	Total Individuals
Speech Delay	49	35
Motor Delay	49	35
Facial Dysmorphisms	42	30
Developmental Delay	37	26
Behavioral Differences	37	26
Intellectual Disability	35	25
Muscular Problems	31	22
Learning Difficulties	30	21
Psychiatric Diagnosis	30	21
Epilepsy	24	17
Microcephaly	20	14
ASD	18	13
Short Stature	14	10
Congenital Heart Condition	11	8
Macrocephaly	7	5

Arranged in descending order of frequency.

**Table 4:** Differences in clinical features in the proband by parental origin and gender of the 15q11.2 BP1-BP2 microdeletion.

		Parent-of-Origin Differences						Gender Differences					
	Clinical Feature	Full Cohort (*N* = 71)		No Siblings (*N* = 55)		No Siblings and/or VOUS (*N* = 44)		Full Cohort (*N* = 71)		No Siblings (*N* = 55)		No Siblings and/or VOUS (*N* = 44)	
		% Mat	% Pat	% Mat	% Pat	% Mat	% Pat	% F	% M	% F	% M	% F	% M
Grouped Clinical Features	Any Behavior	62	41	59	42	50	45	31	64 **	36	61	32	60
	Any Delays	65	68	62	77	54	75	48	79 **	55	79	47	76 *
	Any Non-physical	97	92	97	92	96	95	90	98	91	97	89	100
	Any Physical	56	65	59	73	54	65	55	64	59	70	53	64
Specific Clinical Features	ASD	29	8 *	24	8	21	5	17	19	18	15	11	16
	CHD	0	22 ***	0	19 **	0	10	17	7	14	6	11	0
	DD	41	32	41	38	38	40	24	45	32	45	26	48
	Difficult Behaviors	47	27	45	27	38	25	24	45	27	42	21	40
	Epilepsy	29	19	34	12 *	42	10 **	38	14 *	32	18	32	24
	Facial Dysmorphisms	47	38	48	46	42	45	38	45	45	48	42	44
	ID	44	27	45	31	46	35	28	40	32	42	32	48
	LD	38	22	38	19	38	20	17	38	23	33	16	40
	Macrocephaly	15	0 *	17	0*	13	0	7	7	9	9	5	8
	Microcephaly	15	24	10	27	13	25	17	21	18	18	16	20
	Motor Delay	53	46	48	54	50	55	28	64 ***	32	64 *	32	68 *
	Muscular Diagnosis	24	38	24	50 *	17	55 **	28	33	36	36	32	36
	Psychiatric Diagnosis	32	27	31	23	29	30	17	38	23	30	26	32
	Short Stature	9	19	10	19	13	20	10	17	14	15	16	16
	Speech Delay	56	43	52	54	46	50	38	57	41	61	32	60

Mat: maternal; Pat: paternal; F: female; M; male; Any Behavior: Any behavioral symptoms; Any Delays: speech, motor, or general developmental delays; Any Non-physical: Any non-physical feature noted; Any Physical: Any physical feature noted; ASD: autism spectrum disorder; CHD: congenital heart disease; DD: Developmental delays; ID: Intellectual disability; LD: Learning disorder/difficulties; Muscular Diagnosis: muscle-related phenotypes; Psychiatric Diagnosis: Psychiatric condition diagnosis. VOUS: variant of unknown significance by genetic testing (e.g., microarray analysis). chi-squared test; *p-*values (significance *p* < 0.05); * *p* ≤ 0.05, ** *p* ≤ 0.01, *** *p* ≤ 0.005.

## Abbreviations

ASD	Autism spectrum disorder
ADHD	Attention-deficit hyperactivity disorder
BP1-BP2	Breakpoint 1–Breakpoint 2
CHD	Congenital heart disease
DECIPHER	DatabasE of genomiC varIation and Phenotype in Humans using Ensembl Resources
GTEx	Gene-Tissue Expression Project
OMIM	Online Mendelian Inheritance in Man
POE	Parent-of-origin effect
VOUS	Variant of uncertain (clinical) significance
